# Development and Evaluation of a Loop-Mediated Isothermal Amplification Assay for Diagnosis of* Schistosoma mansoni* Infection in Faecal Samples

**DOI:** 10.1155/2018/1267826

**Published:** 2018-06-14

**Authors:** Ibrahim N. Mwangi, Eric L. Agola, Robert M. Mugambi, Esther A. Shiraho, Gerald M. Mkoji

**Affiliations:** ^1^Center for Biotechnology Research and Development, Kenya Medical Research Institute (KEMRI), P.O. Box 54840, 00200 Nairobi, Kenya; ^2^Institute of Tropical Medicine and Infectious Diseases, Jomo Kenyatta University of Agriculture and Technology (JKUAT), P.O. Box 62000, 00200 Nairobi, Kenya

## Abstract

Human intestinal schistosomiasis is caused by the blood fluke,* Schistosoma mansoni.* With intensified efforts to control schistosomiasis by mass drug administration using praziquantel (PZQ), there is an urgent need to have accessible, quality-assured diagnostic tests for case detection and disease surveillance and for monitoring efficacy of treatment and other interventions. Current diagnostic tools are limited by suboptimal sensitivity, slow turn-around-time, affordability, and inability to distinguish current from past infections. We describe a simple and rapid diagnostic assay, based on the loop-mediated isothermal amplification (LAMP) technology for diagnosis of* S. mansoni* infection in human faecal samples. The LAMP primers used in this assay were previously described and they target a 121-bp DNA repeat sequence in* S. mansoni. *The LAMP assay was optimized at an isothermal temperature of 63°C for 1 hour. The amplified DNA was either visualized under ultraviolet light after electrophoresis or by directly observing the color change after staining the amplicons with CYBR Green dye. The LAMP assay was evaluated against the microscopy-based procedure and the results were analysed using Cohen's kappa coefficient to determine the degree of agreement between the two techniques. The LAMP assay reliably detected* S. mansoni* ova DNA in faecal samples and parasite DNA in amounts as low as 32fg. When the assay was tested for specificity against other faecal-based soil-transmitted helminths (STH), no cross-reactivity was observed. The LAMP assay was superior to the Kato-Katz assay with a 97% specificity; a high positivity score reliably detecting* S. mansoni *and a Kappa Coefficient of 0.9 suggested an exceptional agreement between the two techniques. The LAMP assay developed has great potential for application in field settings to support* S. mansoni* control and elimination campaigns.

## 1. Introduction

Human schistosomiasis is a snail-borne parasitic infection caused by blood flukes in the genus* Schistosoma* and is among the neglected tropical diseases (NTDs) targeted for elimination [1]. It remains a major public health problem in the tropical and subtropical regions of the world, with an estimated 207 million people infected worldwide, mostly children, and another 779 million people being at risk of becoming infected [2].* S. mansoni*, one of the three major* Schistosoma* species responsible for causing human schistosomiasis worldwide, is endemic in much of the sub-Saharan Africa, Madagascar, the Caribbean area, and parts of South America and causes intestinal schistosomiasis [3].

Schistosomiasis control is heavily dependent on chemotherapy, using the only drug now available, praziquantel (PZQ) [4]. Chemotherapeutic intervention rapidly reduces morbidity in infected individuals, infection prevalence, and intensity, and also the number of parasite eggs reaching the environment [5]. Effective schistosomiasis control and elimination efforts must be supported by suitable diagnostic tests for case detection, for reliably evaluating efficacy of chemotherapy, and for disease surveillance in control programs. As schistosomiasis control efforts intensify in the endemic areas (with renewed interest in the control of neglected tropical diseases) and become effective, prevalence and intensities of infection will most likely drastically reduce, and so more sensitive diagnostic tests will become necessary for case detection, especially, in low intensity transmission areas or for evaluating efficacy of chemotherapy and disease surveillance in schistosomiasis control programs [5]. Ideally, the diagnostic test should be simple to perform, inexpensive, rapid, specific, and sensitive enough, to detect low intensity infections, and capable of handling bulk samples at a go. While rapid immunodiagnostic tests based on antibody detection may be useful, they may not distinguish between previous and current infections. Also, the urine-based CCA assay and other antigen detection tests offer greater sensitivity and rapidity and are suitable for analysis of large samples [6, 7]. However these have not been evaluated adequately under different field settings. Microscopy is the gold standard for diagnosis of* S. mansoni* but it is not sensitive enough to detect low intensity infections; it is tedious and cumbersome to perform and requires expertise to make a diagnosis.

Molecular diagnostic tools based on the polymerase chain reaction (PCR) technology offer greater sensitivity and robustness [8–10] but require costly equipment and are not suitable for field use.

Diagnostic tests based on the loop-mediated isothermal amplification (LAMP) technology on the other hand promise to revolutionize infection diagnosis, in particular, as a tool for point of care diagnosis. The LAMP assay is relatively simple to perform and rapid, does not require sophisticated equipment, can easily be adapted for visual detection of amplified products, and can potentially be performed under field conditions [11]. The LAMP technology has several advantages over the conventional PCR-based procedures, and, in particular, it can be performed at isothermal temperatures in the range 60–65°C; it is highly specific, and offers efficiency [12]. Besides, the large amounts of a highly insoluble magnesium pyrophosphate salt produced as a by-product during the LAMP reaction facilitates visual detection of the amplification products, thus further simplifying the procedure, and can potentially be performed under field conditions [11, 12].

LAMP assays have been developed for detection of* S. mansoni* infection in snails [13] and* S. japonicum* eggs [14],* Ascaris lumbricoides *[15], and hookworm [16] infections in faecal samples. The LAMP assay for diagnosis of* S. japonicum* infection was found to be equally or more sensitive than the PCR assay for detection of the parasite infection [14]. LAMP assays have also been developed for detection of African trypanosome parasites [17],* Plasmodium* parasites [18],* S. haematobium* [13], and* Taenia* species [19].

In a previous study, Abbasi et al. [13] described identification of* S. mansoni* DNA in infected snails from early prepatency. In this paper, we describe a LAMP assay based on the amplification of a 121-bp DNA repeat sequence for the diagnosis of* S. mansoni* infection in faecal samples.

## 2. Materials and Methods

### 2.1. LAMP Primers Used

The LAMP primers used to perform the present assay were those previously described by Abbasi et al. [13] and target a 121-bp DNA repeat sequence in* S. mansoni*, and whose sequences are shown in Table 1.

### 2.2. Test Parasite for Assay Development

A laboratory maintained isolate of* S. mansoni* originally obtained from a naturally infected patient from Kisumu, western Kenya, and which is routinely maintained in the laboratory at the Kenya Medical Research Institute (KEMRI) in Nairobi through Swiss albino mice and* Biomphalaria sudanica* snails was used as a test parasite in the development and optimization of the LAMP assay for detection of* S. mansoni* infection in faecal samples.* S. mansoni* cercariae shed by lab infected snails and approximately 100 cercariae were used to infect individual mice through the percutaneous route essentially, as previously described by Smithers and Terry [20]. Four weeks after infection, the mice faecal samples were collected by placing mice in about 50 ml of a normal saline solution in a 500-ml glass beaker at room temperature to induce defecation [21]. The presence of* S. mansoni* eggs in mice faecal samples was confirmed by examining faecal smears prepared according to the Kato-Katz procedure [22], under a compound microscope. The parasite eggs on the faecal smear were then counted, and the number of parasite eggs per gram (epg) of faecal sample was calculated. The* S. mansoni* adult worms were then harvested from the infected mice by perfusion as previously described by Smithers and Terry [20], and the recovered worms were preserved in 95% ethanol. These parasite specimens were then used in the development and optimization of the LAMP assay. Similarly, faecal samples were obtained from school children in Mwea, who had tested positive for* S. mansoni* by the Kato-Katz procedure and whose parents or guardians had given consent for their children to participate in this study. Mwea division is an irrigation area in central Kenya, where schistosomiasis is endemic. The human faecal samples were used in the evaluation of the LAMP assay.

### 2.3. Parasite DNA Extraction

Genomic DNA was extracted from freshly collected mice or human faecal samples using the QIAMP fast DNA stool mini kit from Qiagen (Catalogue No. 51604) following the manufacturer's instructions. Briefly, 0.2 g of stool sample was weighed out and placed in 2 ml tubes. Approximately 1 ml of inhibit EX buffer was added to the tube containing faecal sample followed by 1 min vortexing. The suspension was then heated at 95°C for 5 min. Proteinase K (15*µ*l) was added to a new tube followed by addition of the supernatant then centrifuged at 14,000rpm. Buffer AL (200*µ*l) was added and mixed by vortexing for 15s followed by incubation in a water bath for 10mins at 70°C. 200*μ*l of absolute ethanol was then added followed by thorough mixing. From this mixture, 600*μ*l of the lysate was pipetted into a spin column and centrifuged at maximum speed (14000rpm) for 1 min, and the supernatant was discarded. Buffer (AW1) was added followed by centrifugation at max speed for 1 min then supernatant was discarded. 500*μ*l buffer 2 (AW2) was then added and centrifuged for 3 mins at max speed. The supernatant was then discarded and elution performed using 200*μ*l ATE buffer. DNA extraction for adult worms was done using a modified method previously described by Truett et al. [22]. Extracted DNA was stored at −20°C until being used for subsequent assay.

### 2.4. LAMP Assay Optimization

Optimization of the LAMP assay was done by varying the reaction temperatures as well as varying the concentrations of LAMP primer sets of forward and backward external primers (F3 and B3), and forward and backward internal primers (FIP and BIP), designed to amplify the 121 base pairs repeat sequence in* S. mansoni* (Sm1-7). The final assay optimized conditions were as follows: the final reaction mixture of 25*µ*l contained primers (40 pmol of FIP and BIP and 5 pmol of F3 and B3 outer primers), DNA polymerase, 8 units of* Bst *I large fragment, 1mM dNTPs, 0.8M betaine; 1× reaction buffer (containing 20mM Tris-HCl, pH 8.8, 10mM KCl, 10mM (NH_4_) 2SO_4_, 8mM MgS_4_, and 1% Tween 20), and target DNA from* S. mansoni. *The reaction was incubated at 63°C in a water bath for 1hr.

### 2.5. Amplicon Detection

The amplified DNA was visualized under ultraviolet light at 320nm after electophoresis on 2% standard agarose gel for 1 hour and then photographed in black and white. Direct detection of amplicons in a reaction tube was also done by direct observation of the reaction with unaided eye after addition of 1:10 dilution 1:10 SYBR I Green dye (Invitrogen, Carlsbad, CA) to the amplicon. Under these conditions, the color in the reaction tube changed from orange to green in the presence of positive LAMP amplicons.

### 2.6. Determination of the Sensitivity and Specificity of the LAMP Assay

Specificity of the LAMP assay was carried out using* S. mansoni* specific primers to amplify DNA of other co-occurring soil-transmitted helminths including* Trichuris trichiura*, hookworm, and* Ascaris lumbricoides*.

To determine the sensitivity of the LAMP assay by establishing the lower detection limit, the genomic DNA concentration was determined using a Qubit® followed by a 10-fold serial dilution of the DNA. The successive serially diluted DNA samples were then amplified with LAMP primers to determine assay sensitivity.

### 2.7. Parasite Infection Intensity and Sensitivity Determination

Faecal samples obtained from infected children with varying infection intensities of* S. mansoni* and categorized as low (1-99 epg), moderate (100-399 epg), and high (>400 epg) infection based on schistosome egg counts on the Kato-Katz faecal smears, and according to the WHO criteria [23], were used in this experiment. The ability of the LAMP assay to detect and differentiate “low”, “moderate”, and “high” infection intensities was determined, and the results were compared with those obtained using the Kato-Katz procedure.

### 2.8. Specificity Test

The ability of the LAMP technique to specifically detect* S. mansoni* in faecal samples, and not other helminths such as the soil-transmitted helminths (STH), which often co-occur with* S. mansoni* in the same individual or within the same locality (and whose eggs and/or larval stages also occur in the host faecal samples), was determined, given that other helminths can potentially interfere with the specificity of the LAMP assay for detection of* S. mansoni* in faecal samples. DNA was extracted from faecal samples of individuals infected with* Ascaris lumbricoides,* hookworm, or* Trichuris trichiura* (common parasites that often co-occur with* S. mansoni* in endemic areas), and amplified in the assay using the* S. mansoni* LAMP specific primers.

### 2.9. Comparison of the Results of the LAMP Assay with Those of the Kato-Katz Procedure

Comparisons were made between the results of the LAMP assay and those of the Kato-Katz procedure (which is the standard test for diagnosis of* S. mansoni *infection) using Cohen's kappa coefficient procedure described by Viera [24] to establish the degree of agreement between the two techniques. This was calculated by the formula* K= (OA-AC / (1-AC*), where K is Kappa coefficient, OA is observed agreement, and AC is agreement by chance.

### 2.10. Study Approvals

This study was approved by the Scientific and Ethics Review Unit (SERU) of the Kenya Medical Research Institute.

## 3. Results

### 3.1. Amplification of* S. mansoni* DNA Using LAMP Primers Targeting the 121-bp Repeat Sequence

**Figure 1:**
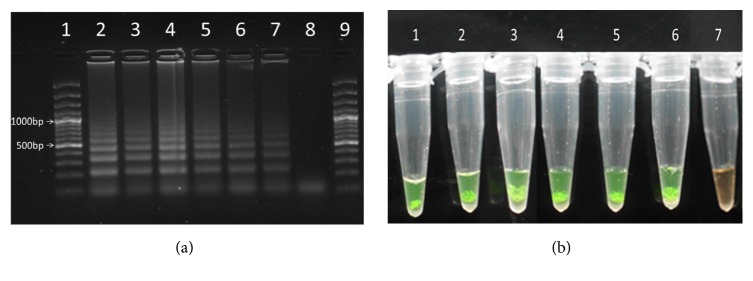
(a**)** Lanes 2 and 3 represent adult worm DNA, lane 4 represents miracidia DNA, lane 5 represents schistosome egg DNA, lanes 6 and 7 represent cercarial DNA, lane 8 represents a –ve control (containing a premix only), and lanes 1 and 9 represent the molecular marker. (b) LAMP amplification reactions in a tube with products stained with SYBR Green I stain, and samples visualized directly with unaided eye. Tubes 1-7 in Figure 1(b) correspond to lanes 2-8, respectively, in Figure 1(a).

The LAMP primers used in this study consistently amplified the 121-bp repeat sequence (ladder like sequence) in the different life cycle stages of* S. mansoni* under the isothermal amplification conditions of 63°C for 60 min as anticipated. These results showed that the developed LAMP assay could successfully and reliably amplify the target 121 pb repeat sequence in* S. mansoni *across all the larval and adult stage of the parasite (Figures 1(a) and 1(b)).

### 3.2. Detection of* S. mansoni* Egg DNA in Human Faecal Samples with Different Parasite Infection Intensities

**Figure 2:**
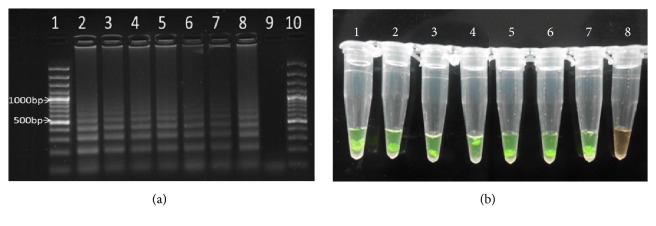
(a) Lane 2 represent* S. mansoni *adult worm DNA (positive control), lanes 3 and 4 represent results of DNA amplification in faecal samples with high intensity* S. mansoni* infection (>400 eggs/g faeces), lanes 5 and 6 represent amplification results of faecal samples with moderate infection intensities (100-399 eggs/g faeces), lanes 7 and 8 represent results of faecal samples with low infection intensities (<100 eggs/g faeces), lane 9 represents amplification results from faecal sample from a non-*S. mansoni* infected subject (negative control), and lanes 1 and 10 represent the molecular marker. (b) LAMP amplification reactions in a tube with products stained with SYBR Green I stain, and samples visualized directly with unaided eye. Tubes 1-8 in Figure 2(b) correspond to lanes 2-9, respectively, in Figure 2(a).

The LAMP assay successfully detected schistosome DNA in human faecal samples with infection intensities ranging between low, moderate, and high intensities based on the WHO criteria of 1-99 eggs per gram (epg) of faecal sample for low infection intensity, 100-399epg for moderate intensity, and > 400epg for high infection intensity. However, the assay could not differentiate between the different categories of infection intensities under the conditions it was performed.  Figure 2(a) shows separation of amplification products on agarose gel after electrophoresis, and Figure 2(b) shows LAMP products in reaction tubes after staining with SYBR Green I dye.

### 3.3. Sensitivity of the LAMP Assay

**Figure 3:**
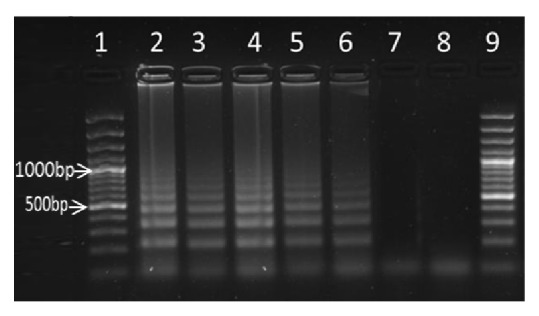
The 10-fold serial dilutions started with 32ng genomic DNA (equivalent to 108* S. mansoni* eggs/g faeces) represented by lane 2 down to 32fg (0.000032ng) represented by lane 6. Lanes 1 and 9 had the molecular marker.

The ability of LAMP technique to detect the lowest amount of* S. mansoni *egg DNA present in a faecal sample was determined after serial dilutions of the sample. Tenfold serial dilutions of total genomic DNA derived from faecal sample obtained from individuals infected with* S. mansoni *was used to test the sensitivity of the LAMP assay. The results of this experiment are presented in Figure 3. It was observed that the lowest detection limit of the LAMP reaction was as low as 32fg of* S. mansoni* egg DNA in a faecal sample. Direct observation of the amplicons in reaction tubes after staining with SYBR Green I dye indicated that all the serially diluted samples were positive for S.* mansoni* DNA because the samples color turned from orange to green, except for those that had less than 32fg of DNA and the negative control sample, as shown on the agarose gel picture in Figure 3.

### 3.4. Specificity of the LAMP Assay

**Figure 4:**
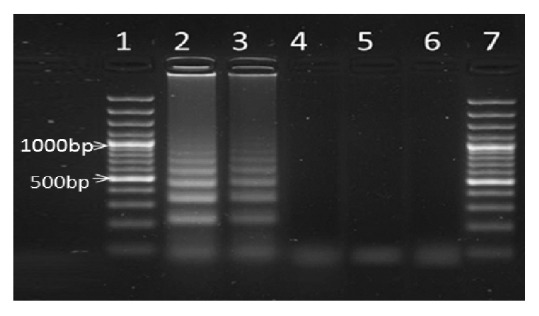
Lanes 2 and 3:* S. mansoni,* lane 4: Hookworm, lane 5: Ascaris, lane 6:* Trichuris trichiura*, and lanes 1 and 7: molecular marker.

When faecal samples containing DNA of* Ascaris lumbricoides,* hookworms, or* Trichuris trichiura* eggs (common parasites that often co-occur with* S. mansoni* in endemic areas) were amplified in the LAMP assay using the* S. mansoni* LAMP specific primers, no amplicons were detected for* A. lumbricoides*, hookworm, or* T. trichiura*, and only samples with* S. mansoni* eggs were amplified.  Figure 4 illustrates the results of this experiment. In other words, only faecal samples from* S. mansoni* infected individuals yielded positive results in the LAMP assay while samples from individuals infected with the soil-transmitted helminths (STH) tested gave negative results in the assay. These results suggest that the assay is specific for* S. mansoni* and will not detect the STH, whose eggs and/or larval stages also commonly occur in human faecal samples.

### 3.5. Comparison of Results from the LAMP Assay with Those from the Kato-Katz Procedure

Out of the 383 faecal samples examined in this study, 176 (46%) samples tested positive for* S. mansoni* infection with the Kato-Katz technique, while only 171 (45%) of the* S. mansoni* positive samples tested positive in the LAMP assay. A total of 207 samples were confirmed to be negative for* S. mansoni* with both the Kato-Katz procedure, and with the LAMP technique. With respect to sensitivity, the LAMP technique scored 97%, whereas, in terms of specificity, it scored 100%. When Cohen's kappa coefficient was determined the result gave a value of 0.9, suggesting that there was an exceptional agreement between the two techniques. In other words, the newly developed LAMP assay could be considered a dependable diagnostic tool for* S. mansoni *infection. The results of Cohen's kappa coefficient determination are summarized in Table 2.

## 4. Discussion

The LAMP assay described in this paper provides a simple, rapid, accurate, and reliable means for detecting* S. mansoni* infection in human faecal samples. While the assay was initially optimized using faecal samples from mice infected with* S. mansoni* under lab conditions, subsequent evaluation of the assay using faecal samples from* S. mansoni* infected school children from an endemic locality in Kenya indicated that it could reliably and consistently detect* S. mansoni* infections in human faecal samples. The assay relies on the amplification of a 121bp repeat sequence present in* S. mansoni* using primers initially developed for detection of* S. mansoni* infections in the snail intermediate host [13]. The primers used in this study were, apparently, capable of amplifying the repeat sequence in all the life cycle stages of the parasite. A LAMP assay was recently developed for early detection of* S. mansoni* infection in rodent faecal samples, and it targeted a sequence corresponding to a mitochondrial minisatellite DNA region [25]. While this assay was found to reliably detect* S. mansoni* DNA in mouse faecal samples, up to levels within 1fg DNA, it would be interesting to see how this assay will perform using human faecal samples. In the assay developed in the present study, the detection limit was found to be 32 fg DNA. Further evaluation of this assay will certainly be necessary.

Although it remains to be established how the present assay will perform using faecal samples collected in areas where schistosomiasis control and elimination are being implemented using chemotherapy; we have found the assay to perform satisfactorily using human faecal samples with infection intensities in all the 3 categories of the WHO criteria of low (1-99 eggs per g (epg) of faeces), moderate (100-399 epg), and high (>400 epg) infection intensities [23]. However, the assay could not differentiate between the different categories of infection intensities.

The LAMP assay results were generally similar to those of the microscopy-based Kato-Katz procedure, with sensitivity and specificity of the two assays being within the same range.

While the microscopy-based Kato-Katz procedure for detection of* S. mansoni* infection is considered cumbersome to use and requires expertise to perform, the LAMP assay is relatively simple to perform and it requires no expertise to perform. It can employ a simple process for detection of amplified products which relies on use of SYBR Green I dye, and the products can then be visualized with a naked eye, making it possible to use the assay under field conditions. Furthermore, the assay can potentially be adapted for analysis of several samples at the same time and is therefore ideal for use in resource-limited settings.

## 5. Conclusion

In spite of the advantages of the LAMP assay over the microscopy-based Kato-Katz procedure, the LAMP assay developed will require further optimization and refinement, before it can be applied routinely for diagnosis of* S. mansoni*. Specifically, the DNA extraction procedure needs to be simplified yet remain efficient and inexpensive. Also, availability of a premix version of the reagents will further increase efficiency and turnaround time. Follow-up studies have been carried out for validation and optimization of LAMP assays for practical use in field laboratories for diagnosis of schistosomiasis [26], but the initial study had shortcomings on the field applicability and practical use scale-up [14].

Although this assay was tested in a closed single-tube, there is concern that contamination of the working area could occur if the reaction tube is opened after amplification. As such, prevention of cross-contamination in the test samples is a priority when performing this assay, and this can be realized by use of highly sterilized apparatus, frequent change of gloves between every assay, and strict separation of reagents used in the assay.

In conclusion, good progress has been made in the development of a LAMP test for detection of* S. mansoni* infection in faecal samples which can potentially also be used to support schistosomiasis control and elimination efforts in the endemic areas. A sensitive and specific diagnostic system for* S. mansoni* infection is critical for precise detection of the infection as well as for evaluation of mass drug administration (MDA) efforts (the WHO recommended schistosomiasis control and elimination strategy) and as a potential tool for disease epidemiological surveillance and in operational research.

## Figures and Tables

**Table 1:** Loop-mediated isothermal amplification primer sets for *S. mansoni *DNA amplification*∗*.

Primer set	Primer position	Primer sequence 5′ 3′
Sm1-7	F3	GAT CTG AAT CCG ACC AAC CG
	B3	AAC GCC CAC GCT CTC GCA
	FIP: F1c + F2	AAATCCGTCCAGTGGTTT **TTTT**
	BIP: B1c +B2	GAAAATCGTTGTATCTCCG CCGAAACCACTGGACGGA** TTTT**
		TATTTTTAATCTAAAACAAAC

*∗* F3: forward outer primer; B3: backward outer primer; FIP: forward inner primer composed of the F1c and F2 primers connected by a TTTT hinge (**bold**); BIP: backward inner primer composed of the B1c and B2 primers connected by a TTTT (**bold**) hinge.

**Table 2:** Contingency table comparing LAMP technique with microscopy-based Kato Katz.

		Kato-Katz (gold standard)		
		+ve	-ve	Totals
LAMP	+ve	171	0	171
	-ve	5	207	212
Totals		176	207	383

Kappa Coefficient: 0.9. Given by (K= (OA-AC) / (1-AC).

## Data Availability

The data used to support the findings of this study are available from the corresponding author upon request.
